# Pilot Study Examining Bed Angles and Heights During Ramped Position Intubation in the Emergency Department

**DOI:** 10.7759/cureus.37104

**Published:** 2023-04-04

**Authors:** Dhimitri A Nikolla, Irtaza Asar, Parker Dalglish, Jestin N Carlson

**Affiliations:** 1 Department of Emergency Medicine, Allegheny Health Network, Erie, USA

**Keywords:** intubation in sitting position, patient position, airway management, emergency airway management, ramped position, endotracheal intubation

## Abstract

Introduction: Ramped positioning during emergent endotracheal intubation has been associated with fewer peri-intubation complications, including a decrease in difficult intubations, esophageal intubations, pulmonary aspiration, and hypoxemia. However, the optimal bed angle and height for ramped position intubation have not been determined. Our objective was to examine the effect bed angle and height in the ramped position may have on laryngeal views during emergent intubation in the emergency department (ED).

Materials and methods: We performed a secondary analysis of prospectively collected quality improvement data on intubations from our ED. All adult medical intubations performed with ramped positioning in the ED over a 24-month study period (September 1, 2020, through August 30, 2022) were eligible. We compared laryngeal views using the percentage of glottic opening (POGO) score between ramp angles (≥30° and <30° from horizontal) and bed heights (relative to the intubator, including xiphoid or above, umbilicus or below, and between xiphoid and umbilicus).

Results: Of the 251 patients intubated during the study period, 201 were intubated in the supine position and 50 in the ramped position. Data forms were completed for 25 patients intubated using ramped position in the ED during the study period. The median ramp angle was 30° (interquartile range (IQR) 25, 40) with 16 (64%) subjects intubated at ≥30° and 9 (36%) subjects at <30°. The median POGO scores for bed angles ≥30° and <30° were 95% (IQR 79, 100) and 90% (IQR 75, 100), respectively. Bed heights varied, with four (16%) intubated at the xiphoid or above height, one (4%) at the umbilicus or below, and 20 (80%) between the xiphoid and umbilicus. The median POGO scores at each position were 95% (IQR 76, 100), 0% (IQR 0, 0), and 95% (IQR 79, 100), respectively.

Conclusion: ED clinicians use a variety of bed angles and heights when intubating in the ramped position. More robust investigations are necessary to determine the optimal bed angle and height for ramped position intubation in the ED.

## Introduction

Peri-intubation adverse events in the emergency department (ED), including hypoxemia, are associated with cardiac arrest [1]. The ramped position is a method of positioning patients during emergent endotracheal intubation that may reduce peri-intubation adverse events [2]. The ramped position is performed by flexing the patient at the hips about 20°-45° so that the upper body is raised above the legs [3]. By raising the upper body, functional residual capacity is improved, facilitating ventilation and oxygenation [4-12]. However, the optimal positioning during ramped intubation to achieve intubation success is unknown [3].

Varying bed angles and heights during ramped position intubation may explain conflicting evidence regarding the effect of ramped position on intubation success in acute care settings [3]. While some studies show improved intubation outcomes in the ED and non-operating room settings, other works have not shown a benefit [2,13-17]. In particular, a randomized intensive care unit-based trial found the ramped position reduced first-attempt success from 85.4% to 76.2% compared to the sniffing position (p = 0.02) [15]. However, none of these works explored the effect of varying bed angles and heights or their interaction during ramped intubation.

Few studies examine differing bed angles or heights during ramped position intubation. In 144 operating room patients intubated with video laryngoscopy in the ramped position and randomized to either the umbilical or nipple bed heights, the umbilical bed height resulted in a shorter laryngoscopy time [18]. However, the bed angle was not measured [18]. Alternatively, increasing ramp angle was associated with increased first-attempt success among 231 ED intubations [13]. However, bed height was not measured [13]. Nevertheless, the relationship between bed angles and heights affected the percentage of glottic opening (POGO) score, time to intubation, and the number of laryngoscopy attempts during 250 simulated direct laryngoscopy intubations by emergency medicine residents and fourth-year medical students [3]. However, the interaction between bed angles and heights during ramped intubation in the ED has not been examined. Therefore, our objective was to study the interaction between bed angle and height on laryngeal views using the POGO score during ED intubations in the ramped position. We hypothesized that higher angles (≥30°) and more extreme bed heights (umbilicus and below or xiphoid and above) would worsen reported POGO scores.

## Materials and methods

We performed a secondary analysis of prospectively collected quality improvement data on intubations from our ED. Intubating clinicians reported data through online and paper case report forms (Appendix). The Allegheny Health Network (AHN) Institutional Review Board reviewed the protocol and determined that the project did not meet the definition of human subject research (#000015120). All adult, non-trauma, non-pregnant, first-attempt intubations performed in the ramped position from our single ED over a 24-month study period (September 1, 2020, through August 30, 2022) were eligible. We recorded the patient's age, weight, body habitus (obese or not as determined by the intubator), and sex, as well as intubator use of direct versus video laryngoscopy. The a priori primary outcome was the POGO defined as the amount of glottic opening vertically visualized by the intubator between the anterior commissure and the interarytenoid notch. Independent variables included bed angle (≥30° and <30° from horizontal) and bed height (head of the bed at the intubator's xiphoid or above, umbilicus or below, and between xiphoid and umbilicus). The bed angle was measured by the intubators using a protractor. The 30° cut-off was chosen as it was used to define ramped intubation in a prior study showing the benefits of ramped position intubation in non-operating room settings [2]. The umbilicus and xiphoid bed heights were chosen since prior simulation work suggested that these heights may be optimal [3].

We present categorical variables as counts with percentages and continuous variables as medians with interquartile ranges (IQR). Normality was determined by the Shapiro-Wilk test, and missingness for each variable was reported. Analysis was performed with R version 4.2.1 (R Foundation for Statistical Computing, Vienna, Austria).

## Results

Of the 251 patients intubated during the study period, 201 were intubated in the supine position and 50 in the ramped position. Paper case report forms, containing the bed angle and height data, were completed for 25 patients intubated using the ramped position in the ED during the study period. The mean age was 67 years (SD 13) (two cases missing), the mean weight was 79 kg (SD 24.6) (two cases missing), and 8 (32%) subjects were obese. Intubators were post-graduate year one for five (20%) subjects, two for 11 (44%) subjects, and three for seven (28%) subjects (two cases missing). Video laryngoscopy was used in 18 (72%) cases.

**Figure 1:**
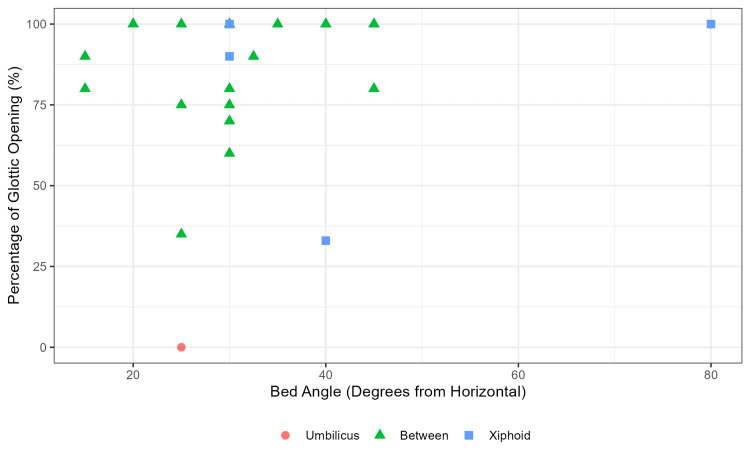
Scatterplot of Percentage of Glottic Opening Scores and Ramped Intubation Bed Angles and Heights Scatterplot of ramped intubation bed angles (x-axis) and percentage of glottic opening scores (y-axis) with bed heights (shape and color).

The median ramp angle was 30° (IQR 25, 40) with 16 (64%) subjects intubated at ≥30° and nine (36%) subjects at <30°. The median POGO scores for bed angles ≥30° and <30° were 95% (IQR 79, 100) and 90% (IQR 75, 100), respectively (Table 1). Bed heights varied, with four (16%) intubated at the xiphoid or above height, one (4%) at the umbilicus or below, and 20 (80%) between the xiphoid and umbilicus. The median POGO scores at each position were 95% (IQR 76, 100), 0% (IQR 0, 0), and 95% (IQR 79, 100), respectively (Table 1). There was no correlation visually between bed angle or height and POGO on a scatterplot (Figure 1). Hypothesis testing was not performed due to the small number of subjects enrolled.

**Table 1:** Percentage of Glottic Opening at Various Bed Angles and Heights *Bed heights were measured using the head of the bed and the anatomical reference points (i.e., xiphoidal and umbilicus) on the intubator. POGO, percentage of glottic opening; IQR, interquartile range

Variable	N (%)	POGO, Median (IQR)
Bed Angle		
≥30° from Horizontal	16 (64%)	95% (79, 100)
<30° from Horizontal	9 (36%)	90% (75, 100)
Bed Height		
Xiphoid or Above*	4 (16%)	95% (76, 100)
Between Xiphoid and Umbilicus*	20 (80%)	95% (79, 100)
Umbilicus or Below*	1 (4%)	0% (0, 0)

## Discussion

We studied a small number of ramped intubations; therefore, definitive conclusions cannot be drawn from our data. Nevertheless, we did not observe a convincing correlation between bed angle or height and POGO score, which differs from our prior work. In our prior study examining various bed angles (25° and 45°) with bed heights (knee, mid-thigh, umbilicus, xiphoid, and nipple/intermammary fold) during 250 simulated intubations performed by emergency medicine residents and fourth-year medical students with direct laryngoscopy, several interactions between bed angle and height were observed [3]. For example, 25° yielded greater POGO scores than 45° at the umbilicus bed height (difference 12% (95% confidence interval, 1, 23)) [3]. However, at the 45° bed angle, the umbilicus bed height had higher POGO scores compared to the xiphoid (difference 20° (7-33)) [3]. Finally, at 25°, the nipple/intermammary fold bed height had more laryngoscopy attempts compared to the xiphoid (0.48 (0.16 to 0.79)) [3].

The interaction between bed angle and height during ramped intubation in the acute care setting has not been published elsewhere; however, bed angle alone has been published. For example, increasing ramp angles (in 5° increments) have been associated with first-attempt success (adjusted odds ratio 1.11 (95% confidence interval, 1.01 to 1.24)) in a cohort of 231 ED intubations [13]. In contrast, upright vs. ramped position was not associated with first-attempt success among 632 ED intubations after propensity score matching (adjusted odds ratio 0.91 (95% confidence interval, 0.44, 1.9)) in a study examining video laryngoscope blade shape in the setting of non-supine ED intubations [19]. The cause of this equipoise between studies is unclear.

In our pilot study, we show that bed heights vary in addition to bed angles during ramped intubation in the ED offering a mechanism for conflicting intubation outcomes between studies [2, 13-17]. This mechanism is supported by a simulation study that suggests that there is an interaction between bed angles and heights during ramped intubation [3]. Therefore, given the variation we observed, the interaction between bed heights and angles during ramped intubation in the ED needs further investigation as it may explain the equipoise between studies [2, 13-17].

Limitations

In addition to low enrollment, our study has some other limitations. It is an observational study from a single center exposing the results to confounders and lack of generalizability. In addition, intubators completed the data collection forms exposing the results to observer and recall biases. Furthermore, intubator skill or experience with ramped position intubation was not measured, which may have confounded the results. Finally, 72% of intubations were performed with video laryngoscopy, and our prior work showed no difference in video POGO between any bed angle and height comparison [3]. Therefore, the bed angle and height interaction during ramped position intubation may be affected by laryngoscope type.

## Conclusions

The interaction between bed angle and height during ramped intubation in the ED and its effects on laryngeal views remains unknown. However, a variety of bed angles and heights were used during ED ramped position intubation. Bed angles around 30° and bed heights between the xiphoid and umbilicus were more common. More robust investigations are necessary to determine the optimal bed angle and height for ramped position intubation.

## Figures and Tables

**Figure 2:**
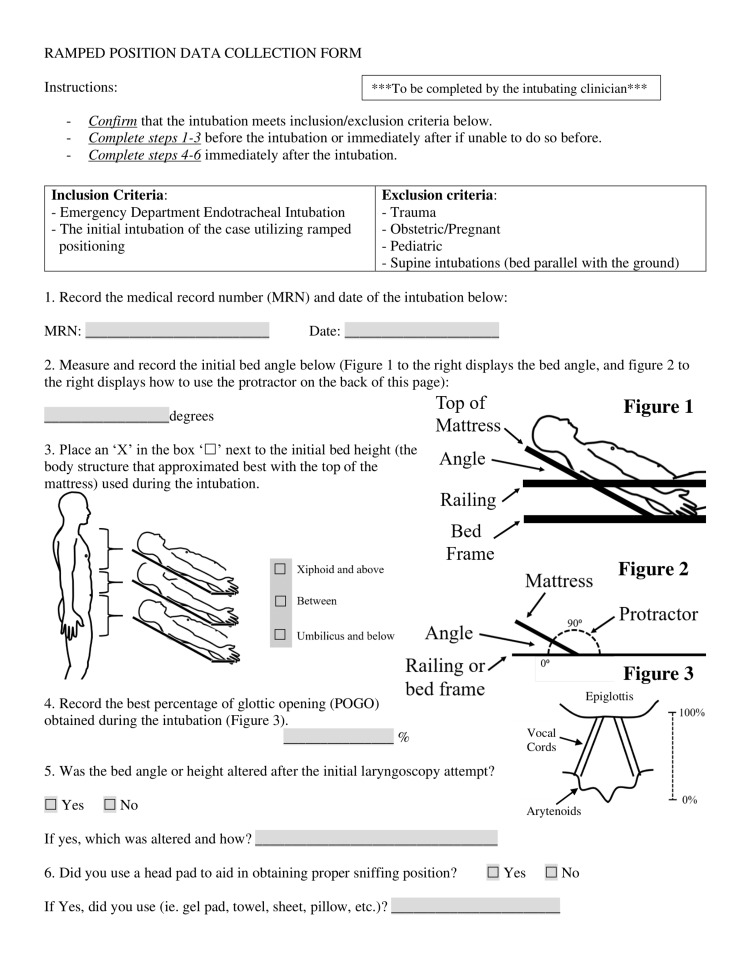
Paper Case Report Form Figure 2 displays the paper case report form used to collect data on bed angle and height.

## References

[1] April MD, Arana A, Reynolds JC (2021). Peri-intubation cardiac arrest in the emergency department: a National Emergency Airway Registry (NEAR) study. Resuscitation.

[2] Khandelwal N, Khorsand S, Mitchell SH, Joffe AM (2016). Head-elevated patient positioning decreases complications of emergent tracheal intubation in the ward and intensive care unit. Anesth Analg.

[3] Nikolla DA, Beaumont RR, Lerman JL, Datsko JS, Carlson JN (2020). Impact of bed angle and height on intubation success during simulated endotracheal intubation in the ramped position. J Am Coll Emerg Physicians Open.

[4] Ibañez J, Raurich JM (1982). Normal values of functional residual capacity in the sitting and supine positions. Intensive Care Med.

[5] Ceylan B, Khorshid L, Güneş ÜY, Zaybak A (2016). Evaluation of oxygen saturation values in different body positions in healthy individuals. J Clin Nurs.

[6] Richard JC, Maggiore SM, Mancebo J, Lemaire F, Jonson B, Brochard L (2006). Effects of vertical positioning on gas exchange and lung volumes in acute respiratory distress syndrome. Intensive Care Med.

[7] Hoste EA, Roosens CD, Bracke S, Decruyenaere JM, Benoit DD, Vandewoude KH, Colardyn FA (2005). Acute effects of upright position on gas exchange in patients with acute respiratory distress syndrome. J Intensive Care Med.

[8] Solis A, Baillard C (2008). Effectiveness of preoxygenation using the head-up position and noninvasive ventilation to reduce hypoxaemia during intubation. Ann Fr Anesth Reanim.

[9] Altermatt FR, Muñoz HR, Delfino AE, Cortínez LI (2005). Pre-oxygenation in the obese patient: effects of position on tolerance to apnoea. Br J Anaesth.

[10] Dixon BJ, Dixon JB, Carden JR (2005). Preoxygenation is more effective in the 25 degrees head-up position than in the supine position in severely obese patients: a randomized controlled study. Anesthesiology.

[11] Lane S, Saunders D, Schofield A, Padmanabhan R, Hildreth A, Laws D (2005). A prospective, randomised controlled trial comparing the efficacy of pre-oxygenation in the 20 degrees head-up vs supine position. Anaesthesia.

[12] Ramkumar V, Umesh G, Philip FA (2011). Preoxygenation with 20º head-up tilt provides longer duration of non-hypoxic apnea than conventional preoxygenation in non-obese healthy adults. J Anesth.

[13] Turner JS, Ellender TJ, Okonkwo ER (2017). Feasibility of upright patient positioning and intubation success rates at two academic EDs. Am J Emerg Med.

[14] Semler MW, Janz DR, Russell DW (2017). A multicenter, randomized trial of ramped position vs sniffing position during endotracheal intubation of critically ill adults. Chest.

[15] Stoecklein HH, Kelly C, Kaji AH (2019). Multicenter comparison of nonsupine versus supine positioning during intubation in the emergency department: a National Emergency Airway Registry (NEAR) study. Acad Emerg Med.

[16] Okada Y, Nakayama Y, Hashimoto K, Koike K, Watanabe N (2021). Ramped versus sniffing position for tracheal intubation: a systematic review and meta-analysis. Am J Emerg Med.

[17] Turner JS, Hunter BR, Haseltine ID (2023). Effect of inclined positioning on first-pass success during endotracheal intubation: a systematic review and meta-analysis. Emerg Med J.

[18] Kang D, Bae HB, Choi YH, Bom JS, Kim J (2022). A prospective randomized study of different height of operation table for tracheal intubation with videolaryngoscopy in ramped position. BMC Anesthesiol.

[19] Nikolla DA, Carlson JN, Stuart PMJ, Asar I, April MD, Kaji AH, Brown CA III (2022). Impact of video laryngoscope shape on first-attempt success during non-supine emergency department intubations. Am J Emerg Med.

